# Predicting the Risk of Malignancy of Lung Nodules Diagnosed as Indeterminate on Radial Endobronchial Ultrasound-Guided Biopsy

**DOI:** 10.3390/jcm9113652

**Published:** 2020-11-13

**Authors:** Sungmin Zo, Sook-young Woo, Seonwoo Kim, Jung Eun Lee, Byeong-Ho Jeong, Sang-Won Um, Hojoong Kim, O Jung Kwon, Ho Yun Lee, Kyungjong Lee

**Affiliations:** 1 Division of Pulmonary and Critical Care Medicine, Department of Medicine, Samsung Medical Center, Sungkyunkwan University School of Medicine, Seoul 06351, Korea; aboutsweets@gmail.com (S.Z.); myacousticlung@gmail.com (B.-H.J.); sangwonum@skku.edu (S.-W.U.); hjk3425@skku.edu (H.K.); ojkwon@skku.edu (O.J.K.); 2 Statistics and Data Center, Samsung Medical Center, Seoul 06351, Korea; wsy.woo@samsung.com (S.-y.W.); seonwoo.kim@samsung.com (S.K.); 3 Division of Nephrology, Department of Medicine, Samsung Medical Center, Sungkyunkwan University School of Medicine, Seoul 06351, Korea; jungeun34.lee@samsung.com; 4 Department of Radiology and Center for Imaging Science, Samsung Medical Center, Sungkyunkwan University School of Medicine, Seoul 06351, Korea

**Keywords:** radial EBUS, lung nodule, nomogram

## Abstract

The next diagnostic step in cases of indeterminate radial probe endobronchial ultrasound (radial EBUS)-guided biopsy results remains uncertain. This study aimed to identify risk factors for malignancy based on clinical findings, chest computed tomography (CT), and radial EBUS images, and to estimate the risk of malignancy in lung nodules that showed indeterminate radial EBUS-guided biopsy results by constructing a nomogram. This retrospective study included 157 patients with indeterminate results on an initial radial EBUS biopsy performed at the Samsung Medical Center from January 2017 to December 2018, but with a definitive final diagnosis. Medical records, chest CT, radial EBUS images, and the final diagnoses were reviewed. Patients were randomly divided into training and validation sets. Factors related to malignancy were identified through logistic regression analysis, and a nomogram was constructed using the training set and subsequently applied to the validation set. Six factors in univariable and multivariable analyses, including upper lobe location, spiculation, satellite nodules, echogenicity, presence of dots or linear arcs, and patency of vessels and bronchi predicted malignancy. A nomogram was constructed based on these predictors. The area under the curve (AUC) value of the nomogram was 0.858 using the chest CT factors, which improved to 0.952 when radial EBUS factors were added. The calibration curve showed good agreement between the actual and nomogram-predicted malignancy outcomes. The utility of radial EBUS images for revealing risk factors of malignancy was confirmed. Furthermore, our nomogram was able to predict the probability of malignancy in lung nodules with indeterminate radial EBUS-guided biopsy results.

## 1. Introduction

Diagnosing peripheral pulmonary nodules remains a significant challenge for pulmonologists. Several quantitative models have been developed to estimate the pretest probability of malignancy of pulmonary nodules, to expedite treatment of malignant nodules and minimize procedures for benign ones [1]. Classical models for determining the probability of malignancy constructed by the Mayo Clinic [2], the Veterans Association (VA) [3], and Brock University [4] include clinical factors, such as age, smoking history, cancer history, and radiological characteristics on chest computed tomography (CT), such as the diameter and location of the nodule. Many attempts have been made to identify risk factors for malignancy of pulmonary nodules, which may inform the next diagnostic step.

With the detection of pulmonary nodules rising, this has led to improve diagnostic sensitivity of advanced bronchoscopic techniques, including radial probe endobronchial ultrasound (radial EBUS) [5]. In a meta-analysis performed in 2017, the overall diagnostic yield for radial EBUS was 70.6% [6].

Apart from its role in improving the bronchoscopic biopsy yield for peripheral lung nodules, the image findings of radial EBUS provide clues regarding the nature of the lesions [7]. Kurimoto et al. [8] analyzed radial EBUS images by focusing on internal echoes, vascular and bronchial patency, and the morphology of hyperechoic areas, and correlated the results with the histopathologic findings of surgical resection. Their research suggested that radial EBUS images can provide additional information, thus enhancing predictions of the malignancy potential of lesions relative to previously identified clinical and chest CT findings.

However, there are limitations when interpreting radial EBUS-guided biopsy results. In clinical practice, when the biopsy result is “positive for malignancy”, clinicians can manage the nodules as cancer. On the other hand, remaining “indeterminate” results, which may involve atypical cells, fibrosis, and inflammation, do not completely exclude the possibility of cancer. There is no consensus regarding whether invasive procedures, such as biopsy or surgery, or clinical surveillance should be applied in such cases. The best diagnostic approach in cases with indeterminate results has not been determined.

In this study, we hypothesized that radial EBUS image findings may provide useful information for predicting the risk of malignancy in lung nodules and focused on the malignant potential of indeterminate radial EBUS-guided biopsy results. First, along with identifying risk factors for malignancy from clinical findings and chest CT, we also examined radial EBUS images. Second, based on the identified risk factors, we developed a nomogram to estimate the risk of malignancy in indeterminate radial EBUS-guided biopsy results, which enables us to identify patients who may receive greater benefit from additional procedures.

## 2. Material and Methods

### 2.1. Study Patients

We retrospectively reviewed the medical records of 494 patients who underwent radial EBUS biopsy at the Samsung Medical Center, Seoul, Korea from January 2017 to December 2018. Among these patients, 212 initial radial EBUS-guided biopsy results were indeterminate. Eight patients were excluded due to missing radial EBUS images or poor image quality. An additional 47 patients without a definitive diagnosis were excluded, leaving a total of 157 patients included in this study. Definitive diagnoses were those (1) pathologically confirmed as cancerous by surgical specimen analysis, endobronchial ultrasound-guided transbronchial needle aspiration (EBUS-TBNA), a core needle biopsy, or additional radial EBUS, or (2) with culture results of sputum or bronchial washing that revealed tuberculosis or atypical mycobacteria with adequate antibiotics response, or (3) with follow-up chest CT suggesting resolved or improved lesions with reassessment of radiologist confirming the possibility of benign. In these cases, the patients were observed for at least 6 months.

The Institutional Review Board of Samsung Medical Center approved this study and permitted the review and publication of patient records (IRB No. 2019-03-106). The requirement for informed consent was waived given the retrospective nature of the study.

### 2.2. Variables

Demographics, clinical characteristics and final diagnoses were obtained through electronic chart review. The clinical factors included age, sex, cancer history, and smoking history. All chest CT images were reviewed by a pulmonologist and a radiologist to determine the maximum diameter, location (upper lobe), type (solid or sub-solid), and shape (round or complex) of nodules, presence of emphysema with nodules, spiculation, bronchus sign, and presence of satellite nodules. Satellite nodules were defined as small discrete shadows near the main lesion [9]. All radial EBUS image findings were evaluated for the following features: echogenicity (homogeneous or heterogeneous); attenuation (maintenance of echogenicity considering midline as a fiducial line); margin (regular or irregular); shape (round or complex); presence of dots or linear arcs; and patency of vessels and bronchi.

### 2.3. Statistical Analysis

To develop a nomogram for predicting the malignant potential of lung nodules that showed indeterminate biopsy results on radial EBUS, a model was built using a training set and a validation set. For this, independent cohorts were devised by randomly dividing the patients into training (70%) and validation sets (30%).

The nonparametric Mann–Whitney *U* test was used to compare continuous variables, and the chi-square test or Fisher’s exact test was used to compare categorical variables between the benign group and malignant group in the total data and between training set and validation set.

Univariable logistic regression analysis of the training set was used to identify potential predictors of malignancy. Using the variables with the result of *p* < 0.1 from univariable logistic regression, backward selection multivariable logistic regression analysis was used to analyze the associations among potential predictors. Selected factors with statistical significance were used to develop the nomogram.

The ability of the nomogram to discriminate (i.e., distinguish among patients with different outcomes) and calibrate (i.e., the degree of correspondence between predictions and actual outcomes) was quantified [10,11,12]. Discrimination performance was quantified based on the area under the curve (AUC). Calibration performance was measured by plotting the predicted malignancy against the actual outcomes, as tertiles, with 1000 bootstrap resamples [1,13].

In addition, the AUC of the nomogram constructed based only on chest CT factors was compared with that constructed using chest CT and radial EBUS factors, to verify the radial EBUS features as risk factors for malignancy.

All reported p values were two-sided and *p* < 0.05 was considered statistically significant. All analyses were performed using R software (version 3.2.2; R Foundation for Statistical Computing, Vienna, Austria).

## 3. Results

**Figure 1:**
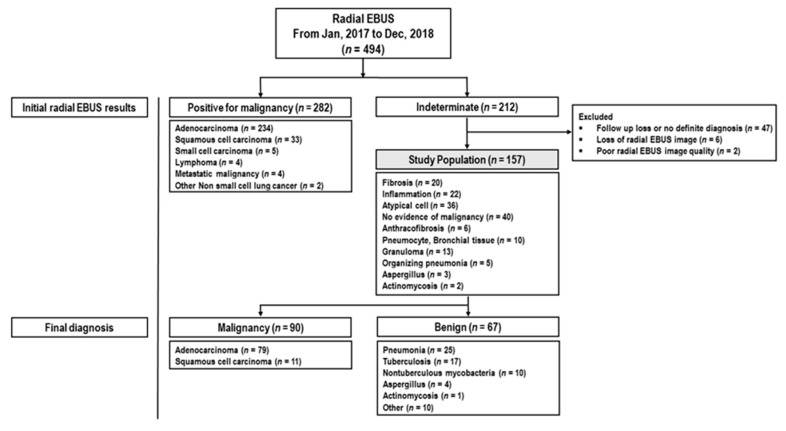
Initial radial EBUS-guided biopsy results and final diagnosis. “Other” includes cryptococcosis, Toxocara canis, sarcoidosis, mucocele, and sarcoidosis. radial EBUS: radial probe endobronchial ultrasound.

Among the 494 patients who underwent radial EBUS biopsy, 157 patients with indeterminate radial EBUS-guided biopsy results were included in this study (Figure 1). The indeterminate results included fibrosis, inflammation, atypical cells, and negative for malignancy. Among the 157 patients, 90 were additionally diagnosed as malignant using other modalities, such as repeat radial EBUS, EBUS-TBNA, a core needle biopsy or surgical specimen analysis. The remaining 67 were diagnosed as benign based on culture results or radiological follow-up for at least 6 months.

The clinical characteristics, chest CT and radial EBUS image features of 157 patients with indeterminate radial EBUS-guided biopsy results are described in Table 1. Overall, 91 (58.0%) males and 66 (42.0%) females, with a mean age of 66.0 years, were included in the study. The prevalence of malignant lung nodules was 57.3%.

In total, 108 (70%) of 157 lung nodules were randomly included in the training set. No significant difference was observed in the clinical, chest CT, or radial EBUS factors between the training and validation sets (Table 2). The clinical characteristics, chest CT, and radial EBUS features of the training set are described in Table 3.

Table 4 summarizes the results of the univariable and multivariable logistic regression analyses of the clinical, chest CT and radial EBUS factors in the training set. The multivariable analysis revealed that chest CT factors, including upper lobe location, spiculation, and absence of satellite nodules were associated with malignancy. Furthermore, malignant nodules tended to be more heterogeneous in terms of echogenicity, had more dots or linear arcs, and had vessels and bronchi that were not patent on radial EBUS images. However, no significant differences were observed in clinical factors between malignant and non-malignant cases.

**Figure 2:**
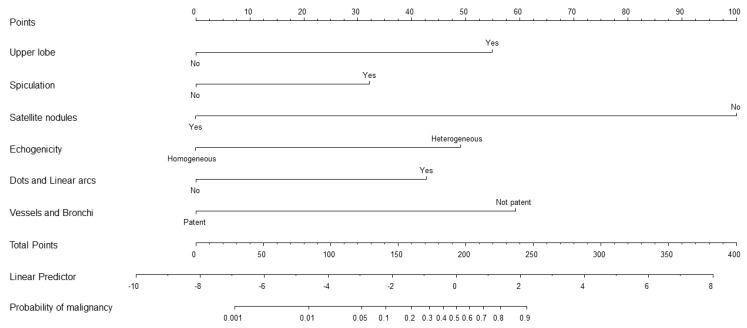
Nomogram for predicting the probability of malignancy. Nomogram for predicting the probability of malignancy of pulmonary nodules diagnosed as indeterminate on initial radial EBUS-guided biopsy, based on clinical, chest CT, and radial EBUS factors. To obtain the nomogram-predicted probability, chest CT and radial EBUS factors of the patient must be placed on a nomogram scale. Then by drawing a vertical line up to the “Points” axis, corresponding prognostic points are obtained. Using the sum of the point acquired from each factor, another vertical line can be drawn from the “Total points” axis down to the “Probability of malignancy” axis, providing the probability of lung cancer.

A nomogram was constructed using the six factors related to malignancy identified by multivariable regression (Figure 2). Through this nomogram, it became possible to calculate the probability of malignancy of lung nodules diagnosed as indeterminate.

**Figure 3:**
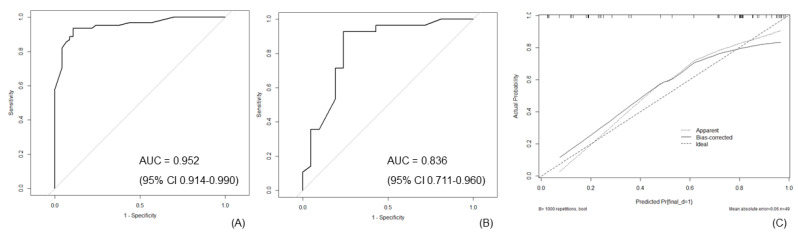
Performance of the nomogram. (**A**) Discrimination performance in training set was high (AUC = 0.952; 95% CI, 0.917–0.990). (**B**) Discrimination performance in validation set was good (AUC = 0.836; 95% CI, 0.711–0.960). (**C**) Calibration curve for the relationship between the predicted and actual malignancy outcomes. The x-axis represents the nomogram-predicted malignancy probability, and the y-axis indicates the actual outcome. The “calibration curve” (dotted line) closely approximates the “logistic calibration curve” (solid line), demonstrating good agreement between the nomogram-predicted and actual malignancy outcomes for indeterminate lung nodules. AUC = area under the curve; CI = confidence interval.

Based on analysis of the training set, the AUC for the nomogram was 0.952 (95% confidence interval [CI] 0.914–0.990) (Figure 3). Based on analysis of the validation set, discrimination performance of the nomogram was good (AUC = 0.836; 95% CI 0.711–0.960). Calibration plots of the training and validation sets indicated good agreement between the predicted and observed outcomes, with close correspondence between the predicted and actual malignancy outcomes.

**Figure 4:**
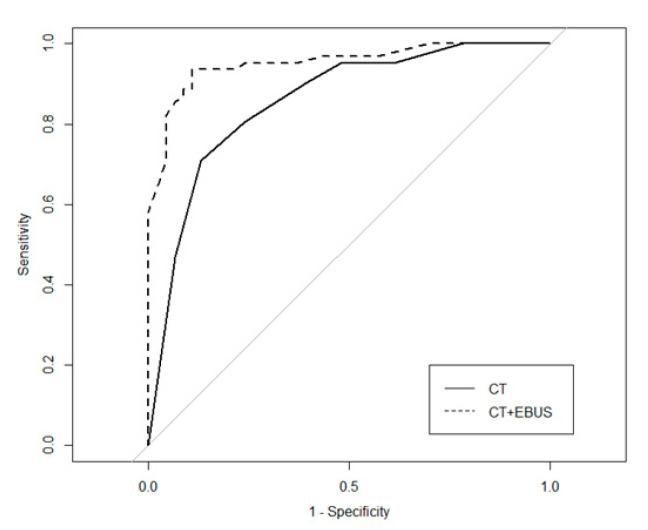
The AUC of the nomogram based only on chest CT factors compared to that based on both chest CT and radial EBUS factors (training set). The ROC curve of the chest CT factors-only nomogram (solid line) and the ROC curve of both chest CT and radial EBUS factors-based nomogram (dotted line). The AUC increased from 0.858 (95% CI, 0.786–0.930) to 0.952 (95% CI, 0.914–0.990) after adding the radial EBUS factors. AUC = area under the curve; CT = Computed tomography; CI = confidence interval; radial EBUS = radial probe endobronchial ultrasound; ROC = receiver operating characteristic.

In addition, as shown in Figure 4, the AUC improved from 0.858 to 0.952 after adding the radial EBUS factors to the nomogram, verifying the role of radial EBUS features as risk factors for malignancy.

## 4. Discussion

Our study was motivated by the lack of definitive guidelines for proceeding in cases with “indeterminate” radial EBUS-guided biopsy results, which do not exclude malignancy. We focused on the clinical utility of radial EBUS images for distinguishing between malignant and benign lesions, which has also been discussed in previous studies [7,8,14].

First, we evaluated the radial EBUS images of pulmonary nodules, as well as clinical findings and chest CT images, to determine diverse risk factors for malignancy. Based on previous radial EBUS image studies [7,8,14], echogenicity, attenuation, margin, nodule shape, the presence of dots or linear arcs, and the patency of vessels and bronchi were evaluated on radial EBUS images. Logistic regression analysis revealed that echogenicity, the presence of dots or linear arcs, and the patency of vessels and bronchi were significantly associated with malignancy.

Echogenicity on radial EBUS images is closely associated with the arrangement of cells and the quantity of fibrous stroma [7]. Several studies [15,16,17,18] on gastrointestinal tract lesions have revealed that heterogeneous echogenicity is more likely to indicate malignancy than homogeneous echogenicity. It is believed that loss of normal tissue and chaotic tumor cell growth with central fibrosis, necrosis, and hemorrhage leads to heterogeneity in the internal structure of lung masses on EBUS images [8]. Dots and linear arcs denote an irregular hyperechoic pattern within lesions, primarily around the radial probe [8]. Kurimoto et al. [8] analyzed radial EBUS images and surgical specimens and found that dots and linear arcs correspond to residual air in the alveoli, which is characteristic of a well-differentiated adenocarcinoma, indicating malignancy. The patency of vessels and bronchi is believed to reflect the degree to which anatomic structures are preserved in cases of lung parenchyma. Tumor cells grow to form a solid mass invading the lung parenchyma in cases of peripheral lung cancer, and the patency of the bronchi seldom remains intact as the tumor increases in volume [18]. Therefore, a lesion with patent vessels and bronchi based on radial EBUS probe is likely to be benign, as in our results.

According to our results, an upper lobe location, spiculation, and an absence of satellite nodules were, withal, the chest CT factors associated with malignancy. An upper lobe location and spiculation of pulmonary nodules are well known independent predictors of malignancy [2,4,19]. The National Lung Screening Trial data set showed 65.7% of all lung cancers were located in the upper lobe [20]. Furthermore, the result of NELSON trial [21], the largest European lung cancer CT screening trial, observed that 64.1% of all lung cancers were localized in the upper lobe. Especially, 45.0% were localized in the right upper lobe. This is a known phenomenon explained by the fact that the airflow at the beginning of the breath is the largest toward the right upper lobe bronchus that eventually causes more deposition of particles in tobacco smoke and their carcinogenic effects in the right upper lobe [22,23]. Spiculation is caused by an obstruction of pulmonary vessels, or by the filling of lymphatic channels with tumor cells; in turn, this results in interlobular septal thickening and fibrosis, and is highly predictive of malignancy (positive predictive value of up to 90%) [24,25]. In contrast, the satellite lesions frequently observed in patients with tuberculoma, or accompanied by organizing pneumonia, tended to be benign in a tuberculosis (TB)-endemic area [26]. In that study, in cases with satellite lesions around a solitary pulmonary nodule, the rate of benign status was 90.9%.

Based on the significant predictors of malignancy in univariable and multivariable analyses, we constructed a nomogram to calculate the probability of malignancy of pulmonary nodules with indeterminate radial EBUS-guided biopsy results. Our results are expected to be useful for patients and clinicians with respect to the decision between an invasive diagnostic procedure or observational follow-up.

The average predictive accuracy regarding the malignancy of pulmonary nodules was reported as 80% using current models based on clinical findings and chest CT factors [2,3]. In our study, the AUC of the nomogram using only chest CT factors was 0.858, but this improved to 0.952 after adding the radial EBUS factors. This confirmed the potential of radial EBUS factors for predicting malignancy.

Our study had several limitations. First, since our cohort comprised only patients with indeterminate radial EBUS-guided biopsy results, it showed a relatively homogeneous nature. For this reason, although several studies [2,3,4] have demonstrated the importance of clinical factors such as age, smoking history, and cancer history for predicting malignancy, these factors were not significantly associated with malignancy in our study. Furthermore, our nomogram could only be applied to patients with indeterminate radial EBUS-guided biopsy results and could not be used in calculating the malignant potential of general patients with pulmonary nodules. Second, even though our nomogram calculated the probability of malignancy of indeterminate radial EBUS-guided biopsy results, precise cut-off values indicating the need for an additional invasive procedure are still lacking; further studies are, therefore, required. British Thoracic Society guidelines [19] suggest patients with a low risk (<10%) of malignancy should undergo CT surveillance. When the risk is moderate-to-high (>10%), additional testing should include fine-needle aspiration, endobronchial ultrasonography, and positron emission tomography. Surgery is recommended for high risk (>70%) patients. Since our study included not only clinical and chest CT factors, but also radial EBUS factors, larger population-based studies should be performed to determine cut-offs specifically for radial EBUS. Third, this study was performed at a single center; further studies including multiple centers and larger populations and controlling of operator factors are needed to validate our results. Fourth, although presence of the satellite nodules was found to be highly predictive of benign pulmonary nodules, this finding was restricted to TB-endemic areas and may not apply elsewhere. Finally, this is a retrospective study that requires prospective validation, and a constructed nomogram must be confirmed through other group of patients with indeterminate radial EBUS-guided biopsy results. For this reason, a further study involving patients that had undergone radial EBUS from 2019 to 2020 in our center, is in progress to apply and validate our nomogram.

In conclusion, we identified six risk factors for malignancy based on analysis of radial EBUS images, as well as clinical findings and chest CT. Thus, radial EBUS may provide additional information to complement other clinical and radiological data. Furthermore, we developed a nomogram to estimate the probability of malignancy in patients with indeterminate initial radial EBUS findings, which may facilitate identification of patients at higher risk for malignancy.

## Figures and Tables

**Table 1:** Baseline characteristics of the patients by malignancy status.

	Total (*n =* 157)	Benign (*n =* 67)	Malignant (*n =* 90)	*p* Value
**Clinical factors**				
Age, y	66.0 (59.0–73.0)	66.0 (58.0–72.5)	66.5 (59.0–73.0)	0.757
Sex				0.384
Male	91 (58.0)	42 (62.7)	49 (54.4)	
Female	66 (42.0)	25 (37.3)	41 (45.6)	
History of malignancy	30 (19.1)	10 (14.9)	20 (22.2)	0.345
History of smoking	69 (43.9)	29 (43.3)	40 (44.4)	1.000
**Chest CT findings**				
Size, mm	30.0 (21.0–41.0)	31.0 (21.5–45.0)	29.5 (21.0–38.0)	0.408
Upper lobe	84 (53.5)	28 (41.8)	56 (62.2)	0.017
Type				0.108
Solid	110 (70.1)	52 (77.6)	58 (64.4)	
Sub-solid	47 (29.9)	15 (22.4)	32 (35.6)	
Emphysema with nodules	27 (17.2)	6 (9.0)	21 (23.3)	0.032
Spiculation	92 (58.6)	22 (32.8)	70 (77.8)	<0.001
Bronchus sign	48 (30.6)	12 (17.9)	36 (40.0)	0.005
Shape				0.817
Round/Oval	33 (21.0)	13 (19.4)	20 (22.2)	
Complex	124 (79.0)	54 (80.6)	70 (77.8)	
Satellite nodules	47 (29.9)	37 (55.2)	10 (11.1)	<0.001
**Radial EBUS findings**				
Echogenicity				<0.001
Homogeneous	90 (57.3)	52 (77.6)	38 (42.2)	
Heterogeneous	67 (42.7)	15 (22.4)	52 (57.8)	
Attenuation	59 (37.6)	18 (26.9)	41 (45.6)	0.026
Margin				0.017
Regular	22 (14.0)	15 (22.4)	7 (7.8)	
Irregular	135 (86.0)	52 (77.6)	83 (92.2)	
Shape				1.000
Round/Oval	71 (45.2)	30 (44.8)	41 (45.6)	
Complex	86 (54.8)	37 (55.2)	49 (54.4)	
Dots or linear arcs	60 (38.2)	10 (14.9)	50 (55.6)	<0.001
Vessels and bronchi				<0.001
Patent	28 (17.8)	23 (34.3)	5 (5.6)	
Not patent	129 (82.2)	44 (65.7)	85 (94.4)	

Data are presented as number (%), median (interquartile range). Abbreviations: CT = Computed tomography; Radial EBUS = radial probe endobronchial ultrasound.

**Table 2:** Baseline characteristics of the patients in the training and validation sets.

	Training Set (*n =* 108)	Validation Set (*n =* 49)	*p* Value
**Final diagnosis**			1.000
Benign	46 (42.6)	21 (42.9)	
Malignant	62 (57.4)	28 (57.1)	
**Clinical factors**			
Age, y	66.5 (59.0–73.0)	66.0 (58.0–73.0)	0.872
Sex			0.464
Male	60 (55.6)	31 (63.3)	
Female	48 (44.4)	18 (36.7)	
History of malignancy	22 (20.4)	8 (16.3)	0.705
History of smoking	45 (41.7)	24 (49.0)	0.495
**Chest CT findings**			
Size, mm	30.5 (22.0–40.5)	26.0 (19.0–43.0)	0.374
Upper lobe	61 (56.5)	23 (46.9)	0.348
Type			0.660
Solid	74 (68.5)	36 (73.5)	
Sub-solid	34 (31.5)	13 (26.5)	
Emphysema with nodules	17 (15.7)	10 (20.4)	0.624
Spiculation	63 (58.3)	29 (59.2)	1.000
Bronchus sign	37 (34.3)	11 (22.4)	0.193
Shape			0.352
Round/Oval	20 (18.5)	13 (26.5)	
Complex	88 (81.5)	36 (73.5)	
Satellite nodules	34 (31.5)	13 (26.5)	0.660
**Radial EBUS findings**			
Echogenicity			0.401
Homogeneous	59 (54.6)	31 (63.3)	
Heterogeneous	49 (45.4)	18 (36.7)	
Attenuation	43 (39.8)	16 (32.7)	0.496
Margin			0.191
Regular	12 (11.1)	10 (20.4)	
Irregular	96 (88.9)	39 (79.6)	
Shape			0.643
Round/Oval	47 (43.5)	24 (49.0)	
Complex	61 (56.5)	25 (51.0)	
Dots or linear arcs	43 (39.8)	17 (34.7)	0.664
Vessels and bronchi			0.914
Patent	20 (18.5)	8 (16.3)	
Not patent	88 (81.5)	41 (83.7)	

Data are presented as number (%), median (interquartile range). Abbreviations: CT = computed tomography; Radial EBUS = radial probe endobronchial ultrasound.

**Table 3:** Baseline characteristics of the patients in the training set by malignancy status.

	Benign (*n =* 46)	Malignant (*n =* 62)	*p* Value
**Clinical factors**			
Age, y	65.0 (56.0–71.0)	67.5 (59.0–73.0)	0.469
Sex			0.446
Male	28 (60.9)	32 (51.6)	
Female	18 (39.1)	30 (48.4)	
History of malignancy	8 (17.4)	14 (22.6)	0.674
History of smoking	19 (41.3)	26 (41.9)	1.000
**Chest CT findings**			
Size, mm	30.0 (22.0–42.0)	30.5 (22.0–40.0)	0.918
Upper lobe	20 (43.5)	41 (66.1)	0.031
Type			0.037
Solid	37 (80.4)	37 (59.7)	
Sub-solid	9 (19.6)	25 (40.3)	
Emphysema with nodules	4 (8.7)	13 (21.0)	0.143
Spiculation	16 (34.8)	47 (75.8)	<0.001
Bronchus sign	7 (15.2)	30 (48.4)	0.001
Shape			1.000
Round/Oval	9 (19.6)	11 (17.7)	
Complex	37 (80.4)	51 (82.3)	
Satellite nodules	28 (60.9)	6 (9.7)	<0.001
**Radial EBUS findings**			
Echogenicity			<0.001
Homogeneous	35 (76.1)	24 (38.7)	
Heterogeneous	11 (23.9)	38 (61.3)	
Attenuation	14 (30.4)	29 (46.8)	0.129
Margin			0.139
Regular	8 (17.4)	4 (6.5)	
Irregular	38 (82.6)	58 (93.5)	
Shape			0.167
Round/Oval	16 (34.8)	31 (50.0)	
Complex	30 (65.2)	31 (50.0)	
Dots or linear arcs	6 (13.0)	37 (59.7)	<0.001
Vessels and bronchi			<0.001
Patent	16 (34.8)	4 (6.5)	
Not patent	30 (65.2)	58 (93.5)	

Data are presented as number (%), median (interquartile range). Abbreviations: CT = computed tomography; Radial EBUS = radial probe endobronchial ultrasound.

**Table 4:** Univariable and multivariable logistic regression analyses of the training set.

	Univariable Analysis		Multivariable Analysis	
	OR (95% CI)	*p* Value	OR (95% CI)	*p* Value
**Clinical factors**				
Age, y	1.01 (0.98–1.04)	0.601		
Sex (Male)	0.69 (0.32–1.49)	0.339		
History of malignancy	1.39 (0.53–3.64)	0.509		
History of smoking, Ever vs. never	1.03 (0.47–2.23)	0.948		
**Chest CT**				
Size, mm	1.00 (0.97–1.03)	0.853		
Upper lobe	2.54 (1.16–5.57)	0.020	7.81 (1.71–35.75)	0.008
Type (Solid)	2.78 (1.14–6.75)	0.024		
Emphysema with nodules	2.79 (0.84–9.19)	0.093		
Spiculation	5.87 (2.54–13.61)	<0.001	5.65 (1.21–26.47)	0.028
Bronchus sign	5.22 (2.03–13.46)	<0.001		
Shape (Complex)	1.13 (0.42–3.00)	0.810		
Satellite nodules	0.07 (0.02–0.19)	<0.001	0.01 (<0.01–0.09)	<0.001
**Radial EBUS**				
Echogenicity (Heterogeneous)	5.04 (2.16–11.77)	<0.001	5.65 (1.11–28.89)	0.037
Attenuation	2.01 (0.90–4.48)	0.088	4.91 (0.88–27.43)	0.070
Margin (Irregular)	3.05 (0.86–10.85)	0.085		
Shape (Complex)	0.53 (0.24–1.17)	0.117		
Dots or Linear arcs	9.87 (3.64–26.74)	<0.001	8.00 (1.27–50.53)	0.027
Vessels and bronchi (Not patent)	7.73 (2.37–25.19)	<0.001	10.62 (1.65–68.29)	0.013

Abbreviations: CT = computed tomography; CI = confidence interval; OR = odds ratio; Radial EBUS = radial probe endobronchial ultrasound.

## Abbreviations

**AUC:** area under the curve; **CT:** computed tomography; **EBUS-TBNA:** endobronchial ultrasound-guided transbronchial needle aspiration; **Radial EBUS:** radial probe endobronchial ultrasound; **TB:** tuberculosis.
